# Practice guidelines and clinical risk assessment models: is it time to reform?

**DOI:** 10.1186/1472-6947-11-63

**Published:** 2011-10-18

**Authors:** Nariman Sepehrvand, Firouz Ghaderi Pakdel, Mohammad Hosein Rahimi-Rad, Babak Moosavi-Toomatari, Shahrzad Bazargan-Hejazi

**Affiliations:** 1 Students' Research Committee, Urmia University of Medical Sciences, Iran; 2 Department of Physiology, Urmia University of Medical Sciences, Iran; 3 Department of Internal Medicine, Imam-Khomeini Training Hospital, Urmia University of Medical Sciences, Iran; 4 Department of Psychiatry, Charles Drew University of Medicine and Science, & David Geffen School of Medicine, University of California, Los Angeles, CA 90059, USA

**Keywords:** Practice guidelines, Risk assessment models, Fuzzy Logic, Venous Thromboembolism

## Abstract

**Background:**

Clinical practice guidelines and Risk Assessment Models (RAMs) are some useful tools to bring medical evidences into our daily clinical practice. Despite the improvement over the time, they still have some shortcomings.

**Discussion:**

One of these shortcomings is the arbitrary cutoffs used in these tools to facilitate the decision making process. This problem is to some extent due to the "Black or White" approach of modern medicine in making the decisions, whilst in the real world and our daily practice we used mostly an uncertain approach, which is called recently as "Fuzzy" thinking approach.

**Summary:**

The authors of this article believe that the fuzzy type of thinking may resolve the above mentioned shortcomings of clinical practice guideline or risk assessment models and they tried to discuss about this using an example about Venous Thromboembolism related guidelines and RAMs.

## Background

Clinical practice guidelines are useful decision support tools in the process of carrying medical evidences to the point of practice [1]. An example for an important health system problem which experiences a gap between medical evidences and clinical practice is venous thromboembolism (VTE) [2].

Several practice guidelines have developed and focused on this topic, among which the most important one is the evidence-based VTE prevention practice guideline generated and updated by the American College of Chest Physicians (ACCP) [3]. This guideline proposes clinical decision making regarding identification and screening of thromboprophylaxis according to the presence of risk factors in various patient groups. This method of risk evaluation, underestimates the risk of venous thromboembolism in some cases [4]. However this guideline in a section mentioned the individual patient risk assessment and advocates for the use of clinical judgment in some specific circumstances [3].

Despite development of various Practice guidelines for prevention of Venous Thromboembolism (VTE), it remains underused in most countries [5,6]. Improving clinician's compliance with the guidelines is a complex task and partly rest on improving thrombotic risk-assessment methods [7]. There are several risk assessment tools for VTE prevention, from which the three most notable models were developed by Caprini, Cohen, and Kucher [4,8,9]. These models considered the individualized approach to the VTE risk determination in each patient [3]. These risk assessment models (RAM) consist of a list of exposing risk factors (presenting illness or procedure) and predisposing risk factors (genetic and clinical characteristics), each with an assigned relative risk score [4,10]. As stated in the 8^th ^annual conference of ACCP and the earlier editions of the ACCP guideline, the VTE risk factors are generally cumulative [3,11]. In another words, scores for each risk factor are summed to produce a cumulative score, and is used to classify a patient into one of the four risk categories and determine the onset, intensity, type, and duration of recommended prophylaxis [12].

The 8^th ^ACCP conference on VTE prevention, allocated a section to the aforementioned risk assessment models [3] criticizing these models for being cumbersome, and not validated adequately. On the other hand several studies in the past few years have validated the Caprini risk assessment model and linked the score to the eventual development of clinically relevant VTE events up to 60 days post discharge [13-15]. While the original model put people in 4 groups, the validation studies have shown that for each increase in score, the incidence of VTE rises [13]. At the same time, the important role of these models in developing a computerized decision making support systems for clinicians was discussed. Clinical informatics and electronic health records have the potential to ease application of risk assessment model [7].

Another major critique pointed out by the later edition of ACCP guideline regarding the risk assessment models, is the arbitrary cutoffs for age and duration of surgery [3]. We are aimed in this article to suggest a way to address this critique of ACCP guideline to RAMs.

## Discussion

We (the authors) do not confine this shortcoming of RAMs (i.e arbitrary cutoffs) only to age or the duration of surgery as noted by the ACCP guideline. Many other risk factors such as Body Mass Index (BMI), history of major surgery, Stroke, multiple trauma, hip, pelvic and leg fractures in one month prior to hospitalization may have such problem in determining the total VTE risk of an individual.

An example may clarify the issue: a 61 years old man with a history of major surgery of cholecystectomy in the past year is admitted to your hospital with a progressive stroke. Also, another patient, a 74 years old woman with a history of Coronary Artery Bypass Graft (CABG) in the past year, is admitted with a mild Transient Ischemic Attack (TIA). The point score for both two patients using the Caprini Risk Assessment model is 8 and includes 1 for the history of major surgery, 2 for age, and 5 for stroke during the month prior to hospitalization for the first patient, and 2 for age, 4 for history of CABG, and at least 2 for admission with TIA for the second one. The patients acquired equal scores for their ages, but are 61 and 74 years old equal in predisposing VTE? According to the Caprini model, patients between 41-60 years old are assigned one score, and patients between 61-74 years old are assigned 2 score for VTE risk [4]. What is the real difference between 59 and 61 years old, which 1 was assigned for one of them and 2 was assigned for the other? This problem is due to mentioned arbitrary cutoff points which were addressed in the 8^th ^ACCP practice guideline too. How were these cutoff points determined? The cutoff points for age, length of surgery, and BMI were determined over the past 25 years and have solid bases in the literature [11,16]. Also several studies have validated these data (cutoff points which were applied in risk assessment models) and confirmed the use of them to predict the 60 day clinically relevant VTE event [13-15].

Cutoff points are the corner stones and also the flaw of medical guidelines (even evidence-based guidelines). A patient with a body temperature more than 37.2°C axillary in the morning is considered as febrile, but is there a significant difference between 37.1 and 37.3°C, to consider one as febrile and the other one as afebrile? The complex mind of the human does not work like this (with a binary pattern) during its daily interpretations or decision makings.

The authors of this article believe that, this problem of risk assessment models (i.e. arbitrary cutoffs) can be corrected using fuzzy logic in decision making process.

Fuzzy logic which was first introduced by Lotfi A. Zadeh in 1965, transcends the "black and white" approach of the Aristotelian logic, and tries to capture the wide grey areas of imprecision in between [17]. This logic provides a mathematical approach to interpret the grey zones of imprecision.

Considering the imprecise nature of the individual's personal and physical characteristics, Lotfi A. Zadeh anticipated that medicine probably would be the main domain for the application of his theory [18]. But despite this prominent forecast, its progression in the field of medicine remained slow [19].

In recent years interesting proposals for the application of fuzzy logic in medical sciences have been appeared in the literature [20-22], and in this regard a special mention has to be made to the contributions coming from the group of Helgason, aiming for example to better individualize diagnostic process [23], or prescribing and dosing of particular medications at the bedside [24,25].

A new approach to medicine which has had a rapid and continuing growth in the field is the practice of evidence-based medicine (EBM) [26]. The ACCP guidelines for VTE prophylaxis were provided by this evidence-based approach. Some authors consider EBM and Fuzzy logic as two sides of a same coin. The authors of this article believe that fuzzy logic approach is not in contrast to the evidence-based medicine approach, but it is a complementary tool for a more realistic approach to the practice of evidence-based medicine. Fuzzy logic by capturing the grey zone in medical decision makings increases the reliability of risk assessment models. The probabilistic approach of the Fuzzy logic amplifies the applicability of electronic alerts and computerized decision-support systems.

Medical guidelines and risk assessment models capture clinical findings (history or physical examination) and para-clinical findings [12] in the process of determining medical decision. However, as was mentioned above, most of these findings in reality cannot project a binary (black or white) approach. Consider "Swollen legs" in a physical exam: In fuzzy manner of thinking, every body has "swollen legs" to some degree. This degree of membership is almost zero for normal and healthy people, and maybe near 1 for patients with 4+ pitting edema in legs.

ACCP guideline declared "there is little formal understanding of how the various risk factors interact in a quantitative manner to determine the position of each patient along a continuous spectrum of thromboembolic risk"[3]. Indeed, this is a particular case of fuzzy thinking model. In fuzzy manner of thinking, the risk of VTE rest on a continuum. The input from risk assessment models (or even practice guidelines) could be fuzzified for variables that are compatible with this way of thinking. In fuzzy logic, partial antecedents result in partial implications. As a consequence, the output of these risk assessment models will be a fuzzy variable with a degree of membership in each of two neighbor options. Then the outcome (output) of this system could be defuzzified to identify which option is more preferable.

By merging and concurrent use of these two approaches (EBM and Fuzzy logic) in the process of decision-making, fuzzy logic can yield its suitable place in the field of medicine, considering the rapidly progression of evidence-based medicine in this field.

It seems that lack of adequate validity studies, limited number of thromboprophylaxic options, or the complexity of using these risk assessment models for physicians is not enough to burying risk assessment models, and advocating for the simple approach (risk of each patient group) used by ACCP guideline. Fuzzy-based computerized decision support systems can resolve most of the ACCP guidelines shortcomings.

Patients with lower levels of VTE risk may be more affected by using the fuzzy manner of thinking, because among these patients, the application of fuzzy logic may alter the risk level and the options of thromboprophylaxis due to change in cumulative risk score.

The total risk score of patients with higher risk levels (risk score > 7 in Caprini model) may be decreased to some extent using the fuzzy approach, but the option of thromboprophylaxis is similar to when the fuzzy logic is not applied.

As discussed above, a reform in the field of risk assessment tools is needed to enhance their applicabilities in accordance with the expectations of clinicians, as the natural fuzzy thinking systems.

## Summary

The application of Fuzzy thinking model in developing decision support systems can resolve most of the shortcomings of risk assessment models and practice guidelines.

## List of Abbreviations

RAM: Risk Assessment Model; ACCP: American College of Chest Physicians; VTE: Venous Thromboembolism; EBM: Evidence-Based Medicine; BMI: Body Mass Index; TIA: Transient Ischemic Attack; CABG: Coronary Artery Bypass Graft

## Competing interests

The authors declare that they have no competing interests.

## Authors' contributions

NS developed the idea and design of the study, involved in developing the VTE electronic alert software, and helped in writing the manuscript. FG guided the project of developing the computational model for VTE risk assessment. MR contributed in the part related to VTE prophylaxis concept. BM participated in developing the fuzzy-based VTE electronic alert program (software programming), and SB involved in drafting the manuscript. All authors read and approved the final manuscript.

## Pre-publication history

The pre-publication history for this paper can be accessed here:

http://www.biomedcentral.com/1472-6947/11/63/prepub
